# Action sequencing in the spontaneous swimming behavior of zebrafish larvae - implications for drug development

**DOI:** 10.1038/s41598-017-03144-7

**Published:** 2017-06-09

**Authors:** Tobias Palmér, Fredrik Ek, Olof Enqvist, Roger Olsson, Kalle Åström, Per Petersson

**Affiliations:** 1 Integrative Neurophysiology and Neurotechnology, NRC, Department of Experimental Medical Sciences, Lund University, Lund, Sweden; 2 Mathematics, Centre for Mathematical Sciences, Faculty of Engineering, Lund University, Lund, Sweden; 3 Chemical Biology & Therapeutics, Department of Experimental Medical Sciences, Lund University, Lund, Sweden; 4 Department of Signals and Systems, Chalmers University of Technology, Gothenburg, Sweden

## Abstract

All motile organisms need to organize their motor output to obtain functional goals. In vertebrates, natural behaviors are generally composed of a relatively large set of motor components which in turn are combined into a rich repertoire of complex actions. It is therefore an experimental challenge to investigate the organizational principles of natural behaviors. Using the relatively simple locomotion pattern of 10 days old zebrafish larvae we have here characterized the basic organizational principles governing the swimming behavior. Our results show that transitions between different behavioral states can be described by a model combining a stochastic component with a control signal. By dividing swimming bouts into a limited number of categories, we show that similar types of swimming behavior as well as stand-stills between bouts were temporally clustered, indicating a basic level of action sequencing. Finally, we show that pharmacological manipulations known to induce alterations in the organization of motor behavior in mammals, mainly through basal ganglia interactions, have related effects in zebrafish larvae. This latter finding may be of specific relevance to the field of drug development given the growing importance of zebrafish larvae in phenotypic screening for novel drug candidates acting on central nervous system targets.

## Introduction

In natural behavior, motor patterns are arranged into longer action sequences with different compositions depending on factors like the internal brain state of the subject, the behavioral context of the motor act or the functional goal of the behavior. It is not known how such action sequences are constructed and controlled by the nervous system but neuronal circuits of the basal ganglia are thought to play an important role^1–3^. Support for this notion comes for example from studies of locomotive behavior in fish where supraspinal structures involved in controlling different aspects of swimming behavior have been studied in some detail. In these investigations, a regulatory function of the basal ganglia has been identified upstream of regions in the mesencephalon and the more caudal brainstem, which in turn strongly influence locomotor output through descending pathways to spinal cord locomotor circuits (see e.g. refs 4–8). The basal ganglia are known to be highly evolutionary conserved across vertebrate species, and may in fact even have homologies with brain structures in arthropods. Interestingly, neurochemical similarities have also been established among different vertebrates. For example, the well-known modulatory role of monoamines in basal ganglia circuits in mammals has been shown to have a counterpart in fish^5, 9, 10^. Consequently, many of the basic anatomical, neurophysiological and neurochemical features of the central control of locomotor behavior^11^ appear to be very much alike across vertebrate species.

In mammals, natural behaviors typically involve smooth transitions between different motor patterns making it experimentally very challenging to analyze the higher order organization and sequencing of different movement components in any greater detail. In this respect, the locomotor patterns of fish larvae offers a special advantage, as their swimming behavior is highly discretized into isolated relatively brief bouts of locomotive activity. Thus, in order to study different patterns of action sequencing, we have here explored the discretized swimming behavior in zebrafish larvae. For this purpose we have developed an automated image analysis system that lets us analyze the spontaneous swimming behavior in larger groups of larvae in parallel with a level of detail that has previously only been possible in smaller groups using existing motion tracking systems^12–16^. Using this technology platform, we have here studied the swimming behavior in larvae at post fertilization day 10 (dpf 10) and show that the discretized locomotor behavior in fact has a higher order organization which is characterized by the sequencing of swimming bouts of certain types. We also show that pharmacological interventions that are known to alter the sequencing of motor behavior in mammals^17, 18^, in part through interference with basal ganglia circuits^19, 20^, have an analogous dose-dependent effect in zebrafish larvae.

## Results

### A novel method for automated tracking of spontaneous swimming

**Figure 1:**
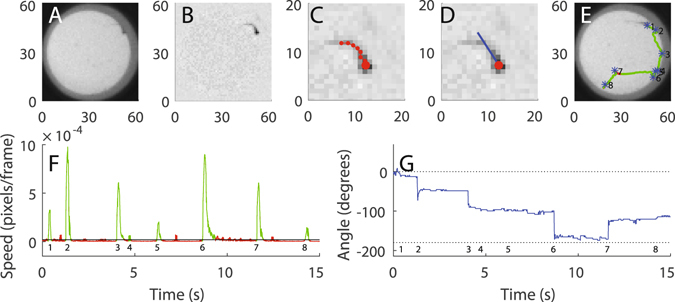
Automated procedures reveal detailed kinematics of the highly discretized swimming behavior. (**A**) Example image frame from high-speed video recording of spontaneous swimming behavior. Each of the 48 larvae, recorded in parallel, is kept isolated in a 15 mm well (red pixels overlaid in the gray scale image denote the body of the larva as automatically classified by the system). (**B**) The corresponding image as shown in (**A**) after background subtraction of pixels lacking temporal dynamics. (**C**) Close-up of the foreground image with superimposed tracking data, where the large dot marks the putative head. Note the improved contrast compared to (**A**) following background subtraction. **(D)** Estimated ﻿gross body orientation in the horizontal plane generated by the system is marked by overlaid﻿ blue line. (**E**) Example episode of spontaneous swimming illustrated by the tracked trajectory of the head position during a 15 s time interval (first frame in the sequence is shown as background). The trajectories of single swimming bouts are colored in green. The blue asterisks represent the starting point of each detected bout during the 15 s period (numbered from 1 to 8; red pixels mark a section of the trajectory where movement speed was below the threshold used for classification of swimming behavior and thus not considered part of a bout). (**F**) Swimming speed plotted as a function of time for the 15 s period shown in (**E**), [numbering of individual bouts as in (**E**); only swimming episodes with a speed above threshold level (black line) above a minimum duration were classified as bouts (#1–8)]. Note that there are some time intervals that are not classified as swim bouts even though the estimated movement speed is larger than the threshold. This is because of the removal of intervals shorter than 0.03 s. (**G**) Allocentric head angle (East = 0 degrees) plotted as a function of time during the 15 s period shown in (**E**). Bout numbering as in (**E,F**).

Swimming behavior was first characterized based on a 65**-**min recording in darkness (using IR-illumination which is invisible to the larvae) in 10 days old zebrafish larvae. In the recording session, 48 larvae were placed in two polystyrene 24-well plates and greyscale digital images were captured at 300 frames per second with a spatial resolution of ∼16 pixels per mm^2^. For every image, the curvature and position of the body was estimated through a two-step process. First, the area corresponding to the 2D projection of the body in the horizontal plane was estimated based on an algorithm classifying foreground objects from the stationary background by fitting of a bimodal Gaussian distribution of light intensities for each pixel throughout the recording period. Second, eight coordinate points spanning from head to tail tip, were fitted to the foreground area through an interpolation procedure based on the pixel intensity of concentric circles centered on the head (Fig. 1A–C *;* see Materials and methods for details). By tracking of the body coordinate positions over a swimming episode, a 2 × 8 × N matrix was generated (where N corresponds to the number of frames collected over the duration of the swimming bout) which captures several detailed aspects of the motor behavior. These data were, in the following analyses, used to produce robust descriptions of the whole-body changes observed in horizontal position (Δr) and head angle (Δψ; Fig. 1D) during the swimming episode. An example of normal swimming behavior as represented by these measures are shown for a 15 s time sequence containing eight discrete swimming bouts in Fig. 1E–G. A highly discretized locomotor behavior is evident in these representations. It is also apparent that although swimming episodes may give the impression of being quite stereotypic on a gross scale, more detailed analyses of speed and angle changes in individual bouts reveal that a wide range of different bout types appear to be present.

### Characterization of individual swimming bouts

**Figure 2:**
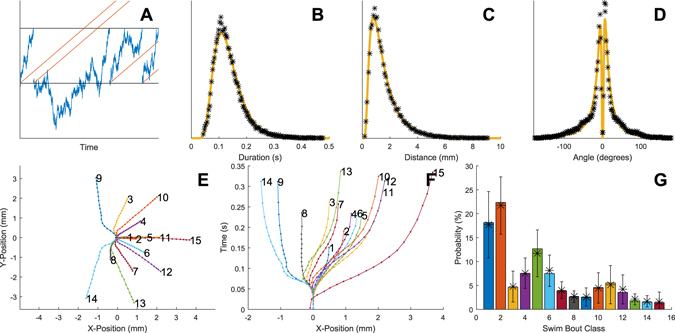
Characterization of swimming bouts based on kinematic features. (**A**) Schematic representation of the underlying assumptions of the Ornstein–Uhlenbeck model. Transitions in behavior occur when the state of the network controlling the behavior passes a certain threshold (upper line). The state of the network is at any point described by a random-walk process which will eventually lead to spontaneous transitions events. In addition, a command signal that actively pushes the state towards the threshold may be present (indicated by the dashed lines). (**B**–**D**) Distributions of kinematic features extracted for each bout (total n = ∼120,000), (**B**) bout duration, (**C**) bout distance and (**D**) turning angle. Yellow lines denote fitting of the Ornstein–Uhlenbeck model to experimental data and asterisks show binning density. (**E,F**) Classification of swimming trajectories reveals differences between swimming behavior observed in individual bouts. (**E**) Trajectories representing the mean trajectories of 15 bout types identified by the algorithm are shown in the horizontal plane (x-y). (**F**) The trajectories in (**E**) shown with time from bout on-set on the y-axis (x-t). The apparent earlier onset of type 15 is caused by data subsampling (see Methods). (**G**) The relative frequency of the number of observed bouts of each type (class code as in E-F; showing that shorter and straight bouts tend to be more common). Whiskers denote mean and SD of the distribution over all individuals – note the relatively small SD and that the pooled probability (bars) closely matches the average over individuals (asterisks) indicating a small inter-individual variance.

High-resolution tracking of swimming behavior in several individuals in parallel makes it possible to quantitatively compare kinematic features in large populations where several thousands of events are analyzed to attain a sensitivity sufficient to reveal minor alterations in motor patterns associated with specific conditions. Building on the developed technology, ∼35,000 swimming bouts recorded in 48 larvae were therefore next analyzed in further detail with respect to their kinematic features. As a first step, distributions of the most elementary kinematic measures, such as bout duration, distance and the cumulative angle change in either turning direction were plotted. These distributions, given the richness of the data sets, could potentially shed some light on aspects of the underlying control mechanisms. For example, if the likelihood of terminating a swimming event is unchanged throughout the execution of a bout (as modelled by a Poisson process where the neuronal network can be thought of as having no memory of when the ongoing bout was initiated) this would result in distributions that are close to exponential. If, on the other hand, the control mechanisms underlying termination of behavior instead resembles a random-walk process (that is, a gradual change in network state as modeled by stepwise movements towards or away from an event threshold denoting the network transition point which causes the termination of the ongoing behavior), distributions will deviate somewhat from strictly exponential distributions and display a relatively higher proportion of long-duration events (typically generated by bouts where the state of the network, as modelled by the random walk, has moved far away from the event threshold). Finally, overlaid on such random processes, motor commands could act to drive the state of the network towards behavioral transition^6, 21^, for example to achieve given behavioral goals. This type of combined process is schematically described in Fig. 2A and can be mathematically modelled as an Ornstein-Uhlenbeck (OU) process^22, 23^. Indeed, fitting an OU model to the observed distance, duration and cumulative turning angle distributions proved to reproduce the observed behavior much better than a Poisson process or a pure random-walk model (Fig. 2B–D and quantitative comparison in Supplementary Table 1). For duration, distance and angle change, respectively the driver parameters in the OU model were found to be 0.0069, 0.0021 and 0.0007/0.0006 (L/R), suggesting that the duration/distance of the swimming bout has a relatively stronger command component than the extent of turning which more resembles a random-walk process. This relatedness to random-walk processes observed for the control of the duration of these motor behaviors is intriguing, since it suggests that the swimming behavior of zebrafish larvae in certain aspect resembles the swimming behavior observed on a much larger scale in several species of adult marine predators^24^. In adult fish, this type of random walk or Brownian motion-like swimming behavior is often complemented with occasional very long straight swimming episodes, referred to as Lévy flights^25^. If present in zebrafish larvae, Lévy flights would however not be detectable in our recording set-up due to the limited size of each well (a necessary restriction to allow for high throughput).

To get a more in depth understanding of the joint translational/rotational behavior during swimming, the bout trajectories were subsequently analyzed in further detail. In agreement with previous reports (e.g. refs 12 and 26) we could confirm, via manual observation of the high-speed video recordings, that a few easily recognizable bout types were present and that some of these were observed much more frequently than others (e.g. scoots and routine turns compared to escape-like swims). However, larvae clearly displayed a much richer repertoire of movements than what could be summarized by only a few bout types. We therefore performed an automated classification of all the swimming trajectories recorded during a 60 minutes long control experiment in 48 larvae where trajectories were described in a coordinate system that was aligned to the starting position/body angle of the larva at the onset of each bout. Dividing the swimming behavior into 15 separate bout classes based on the shape of the trajectory in three dimensions (the two spatial dimensions of the horizontal plane and time) proved to result in a reasonable trade-off between adequate differentiation of observed differences in motor patterns and granularity of the data (Fig. 2E–G; Bayesian information criterion suggested a model order selection in the range of 5–20 classes would be suitable, see Supplementary Figure 1). It should be noted however that these trajectory classes did not represent isolated behavioral types but rather sub-groups sampled from a rather smooth distribution (cf. Supplementary Figure 2; see Material and Methods for details on mathematical procedures and Supplementary Video for 3D-representation of the average trajectory of each class). As expected, some bout classes were much more common than others - in particular short bouts with minor turning (Fig. 2G). It is also worth noting that for the majority of the identified bout classes, a rapid early swimming phase was followed by a gliding phase containing relatively few active motor adjustments, in effect putting rather tight temporal constrains on the time window during which the network state can be actively modulated to control the total duration of the initiated behavior (cf. refs 4 and 27).

### The higher order organization of swimming events

**Figure 3:**
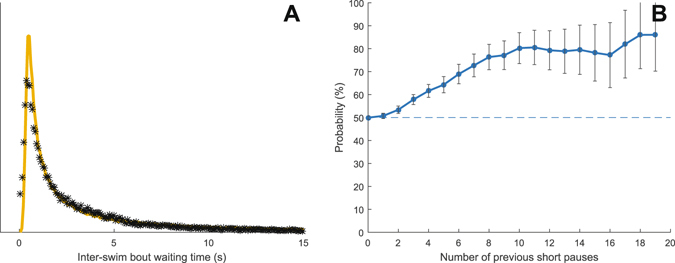
The organization of inter-bout waiting times. (**A**) The distribution of inter-bout waiting times (total n = ∼120,000), yellow line denotes fitting of the Ornstein–Uhlenbeck model to experimental data and asterisks show binning density. (**B**) The probability of observing a short waiting time, given that the previous waiting times were also short increases with the number of similar waiting times observed in a row (short = the shortest 50% of the total amount of waiting times observed in each larva). Error bars denote SEM.

Having analyzed the detailed characteristics of individual bouts and modeled possible state transition mechanisms underlying the termination of motor behavior of each bout we next analyzed the inverse transition – that is, from immobility to locomotion. Also in this case, an OU process proved to model the observed distributions rather accurately (Fig. 3A and Supplementary Table 1). However, compared to transitions from active to passive states the driver component in the OU model that helps controlling the duration of pauses between bouts of activity turned out to be an order of magnitude weaker (0.0001) indicating that neuronal state transitions responsible for initiation of swimming have a relatively stronger stochastic component.

Over more extended time periods, organisms generally cycle through different states of activity/alertness and sometimes display periods of rest^28, 29^. This would result in a change in the duration of stand-stills between bouts extending over a set of bouts. To search for higher order organization of stand-stills between bouts we investigated if larvae had a tendency to switch between either long (top 50% of the total distribution) or short (bottom 50%) waiting-times in a non-random manner. By analyzing the probability of observing waiting-times of the same type (long vs. short) in sequences of waiting-times it became evident that such a mechanism indeed seems to be at play – for example, after observing ten consecutive waiting-times of the same type the probability of switching to the opposite type in the next waiting episode is less than 0.2 (Fig. 3B). Overall however, larvae frequently switch between long and short waiting times and the probability of repeating the previous type of waiting time for any observed consecutive pair of pauses was found to be close to chance level (50%).

Taken together these data suggest that an OU model with a relatively weak drive component can largely reproduce the observed statistical distributions. At the same time, similar waiting-times (long/short) have a tendency to cluster in time. Thus, for a more complete model of the swimming behavior a higher organization of stand-stills between swimming events will eventually have to be incorporated (for example by assuming that the starting point of each random walk in the OU model is determined by a slower secondary process).

### The higher order organization of bout types

**Figure 4:**
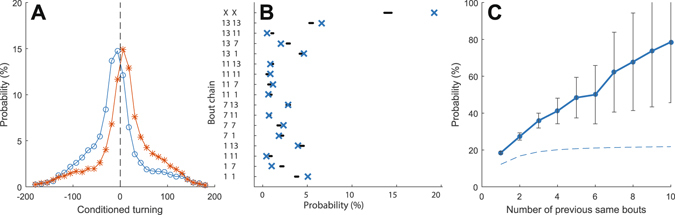
The organization of swimming behavior. Conditioned turning probabilities reveals a tendency to repeat turning in the same direction. Blue curve shows the total turning angle of bouts that directly follow a left turn >10 degrees and red curves the corresponding data for right turns. (**B**) Examples of how different types of bouts are preferentially performed in certain combinations and orders. Blue crosses denoted the actual probability of observing a given bout pair sequence and black dots represent the expected probability based on the overall frequency of observation of the two bout types making up the pair (constructed from 100 shuffled swimming sequences – note the clear separation between the observed randomized data for several of the pairs; The (X-X) sub-chain (top row) is the sum of probabilities for (1-1), (2-2), (3-3), etc.). (**C**) Swimming bouts of a given type tend to be concatenated into longer sequences of repetitive actions. The probability of observing one of the 15 types of swimming bouts is shown as a function of the number of previous observations of the same bout in a row. The theoretical probabilities of the corresponding memoryless system with the same bout class distributions as the actual data are plotted as a dashed line. Error bars represent SEM.

As mentioned in the introduction, a key rationale for the current study was to explore if action sequencing exists also in the comparatively simple locomotor behavior of zebrafish larvae. For example, in a similar way that pauses between swimming events appears to display a higher order organization different types of swimming bouts may also be changing in a state dependent manner. Indeed, Dunn and co-workers recently showed that younger zebrafish larvae (5–9 dpf) often display chains of either left or right turns and suggested that a specific neuronal population in the rhombencephalon may be responsible for this transitory bias towards one of the turning directions^21^. We could here confirm a propensity for repetitive turning pattern also in 10 days old larvae, as shown in Fig. 4A, where the probability of observing a given degree of turning is plotted as a conditioned probability based on the direction of the previous turn (excluding bout pairs where the first turn is <10 degree [which make up ∼15% of the total number of bouts]). This turning bias equates to an observed probability of about 0.65 compared to 0.5 at chance. More importantly however, when we analyzed the probability of observing a repetition of anyone of the 15 bout classes defined above (Fig. 2E,F) we observed an even stronger bias (probability = 0.22 compared to 0.16 at chance). To exemplify the widely differing probabilities of observing action sequences consisting of certain bout types, all permutations of pairs of bouts composed of class 1, 3 and 11 are shown in Fig. 4B. Notably, all types of pairs made up of the same bout type were observed with a probability above chance level whereas several other unique permutations were observed much less frequently than what would be expected based on the probability of observing each of the two components independently. In fact, the tendency to repeat bouts of the same type is even more pronounced in the analysis of longer action sequences. For example if the same type of bout has been repeated ten times in a row the probability of observing the same type of bout out of the 15 possible classes is >0.8 (Fig. 4C *;* chance level is indicated by the dashed line). Control analyses confirmed that the observed tendency for repetitions of bouts was not sensitive to the number of trajectory sample points or bout classes chosen within reasonable limits (Supplementary Figure 3). Finally, although the bout classes defining the different bout types were arbitrarily chosen in our data-set and may not be directly transferable to other data, it is nevertheless interesting to note that certain action sequences are much more commonly observed than others also for some bout pairs involving different bout types (see e.g. 1->11 vs. 11->1 in Fig. 4B).

Hence, we conclude that the swimming behavior of zebrafish larvae appears to be organized into longer sequences of actions where different bouts of motor activity are performed consecutively. In particular, the same type of motor program is frequently concatenated into longer stereotypic action chains - apparently reflecting specific states of the motor control networks.

### Pharmacological manipulations reveal mammalian-like motor control principles

The intricate arrangement of locomotion components revealed in the current analyses, involving parsing of motor behavior into complex sequences, clearly suggests that the seemingly simple swimming behavior of zebrafish larvae most likely contain the basic components needed to allow for comparisons to action sequencing in mammalian species. This finding may have important implications, as zebrafish larvae could therefore become a valuable model system for translational research aimed at developing new therapeutic strategies to treat motor dysfunctions in humans. Following the characterization of normal swimming behavior we therefore next performed a second set of experiments aimed at detecting and describing the changes in motor behavior induced by systemic pharmacological manipulations. To this end, three different drugs which are comparatively well-characterized with respect to their behavioral effects in mammals were evaluated. Two of these drugs were selected to target the monoaminergic system (apomorphine and amphetamine). Monoamines are believed to have a key role in the regulation of goal directed behavior, partly through action on neurophysiological processes of the basal ganglia. Apomorphine is a non-selective dopamine agonist which principally activates dopamine receptors of both the D1 and D2 sub-type. Amphetamine, on the other hand, has a broader effect on monoaminergic signaling but is thought to primarily stimulate the dopaminergic and noradrenergic system by various mechanisms that contribute to an increase in the synaptic levels of these transmitter substances. The third drug, MK-801, instead directly targets the glutamatergic system by acting as a non-competitive antagonist on N-methyl-D-aspartate (NMDA) receptors. This drug is relevant in the context of motor control for the reason that NMDA-antagonists, when administered in lower doses, has been shown in rodents to induce a moderate form of hyperactivity that includes a shift in the pattern of locomotion towards longer uninterrupted bouts of movements (see e.g. refs 30 and 31). All experiments were repeated either two or three times in different groups of animals resulting in a total of 24 or 36 larvae in each treatment group. In the case of apomorphine two different dose regiments were explored based on the previously reported complex dose-response patterns^32^. For MK-801, slightly older larvae were used (15 dpf) since contradictory responses have previously been reported for this drug in younger animals^33, 34^.

**Figure 5:**
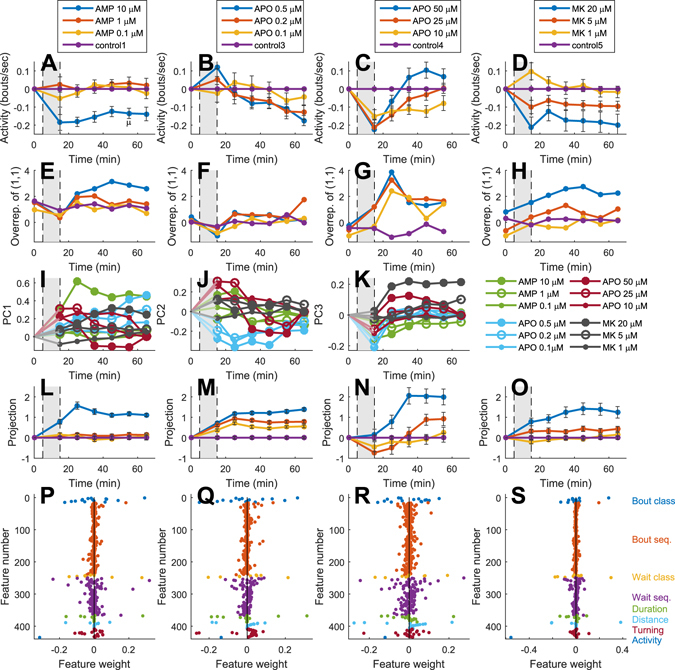
Pharmacological manipulations induce specific changes in swimming behavior involving both simple kinematic features and action sequencing. (**A–D**) Example of pharmacological effects on a single kinematic feature: Changes in the average number of bouts displayed per second following different pharmacological manipulations (shaded area) throughout the recording period reveal drug and dose specific effects. (**E–H**) Example of pharmacological effects on higher order organization of behavior: Changes in the relative overrepresentation of one specific bout pair (type1-type 1, cf. Fig. 2) reveal drug and dose specific effects. Values presented represent the probability (in %) of observing the pair [1, 1] minus the theoretical probability based on the overall observed frequency of the constituent bout type during the same period of time period. (**I–K**) Global changes in swimming behavior shown for each of the treatment groups for the first three dimensions in a PCA sub-space constructed from the larger 435-dimensional feature space. Note the relative separation of the different drug treated groups in certain dimensions and the relative smoothness of the curves indicating gradual rather than abrupt changes in the global motor behavior of each treatment group over time. (**L–O**) Dose dependence of the behavioral changes induced by each drug. The average behavior displayed by the group receiving the highest dose minus the behavior of the control group represents the specific direction and size (=1) of the vector in feature space for each drug. Geometrical projection of the feature vectors obtained for the different doses/time points are plotted for each set of experiments. Note that lower doses generally show intermediate behavioral changes and that all treatment groups show robust effects over time (x-axis represent consecutive non-overlapping sample points from 0 to 65 min). (**P–S**) The relative contribution of the different kinematic features to the global representation of the behavioral changes characterizing each drug. Deviations from zero indicate a difference in this kinematic feature between each drug-treated group and the corresponding control group (e.g. the blue dot with feature #435 marks the reduced activity shown in 5**A–D**). Notably several of the features show clear drug-induced changes. Whiskers denote SEM. [Drug abbreviations: AMP = amphetamine; APO = apomorphine; MK = MK-801].

All drugs resulted in dose dependent changes in swimming behavior. For example, the number of bouts displayed per unit time decreased throughout the experiments, except for high dose apomorphine which showed a more complex biphasic pattern (as previously reported by e.g. refs 32 and 35; Fig. 5A–D). Notably, for MK-801 the observed reduced locomotive activity (swimming distance per bout was also reduced, by approximately 1/3 at the highest dose) clearly contradicts the previously reported effects in rodents and instead bears a resemblance to the motor effects reported in primates^36^. More interestingly, the sequencing of motor behavior was also found to be altered in a drug and dose dependent manner. An example of this is shown in Fig. 5E–H, where the relative overrepresentation of a specific bout pair is plotted as a function of time for the different experiments (i.e. the increased probability of observing that specific bout pair in relation to the expected number of observations based on the overall observation frequency of the constituent bout type, i.e. [P(i, j)–P(i)P(j)]).

Because more complex combinations of kinematic features could be affected by the pharmacological manipulations we next expanded our analyses to include a multivariate approach for evaluating the behavioral changes induced by the different drugs. Accordingly, all the different measures presented above (Figs 1–4), were used to construct a large kinematic space based on a total of 435 features extracted from the swimming behavior (for a complete summary of these 435 features and estimates of their relative interdependency see Supplementary Table 2 and Supplementary Figure 4). First, to get an intuitive understanding of the data, visualizations were constructed showing the global behavioral state of each treatment group as a function of time through principal component analysis (In Fig. 5I–K this is exemplified by the projection of the 435-dimensional state vector onto the first three principal components [explaining 76% of the total variance]). The relatively smooth trajectories showing gradual rather than abrupt state changes between data sets sampled at adjacent time points suggest that this approach generates robust state descriptions. Moreover, the test sensitivity also appears to be good since the different treatment groups are distinguishable even when the space is reduced to only four dimensions. Second, in order to more directly compare the specific effects of individual drugs, dose-response relations were analyzed for each drug. To this aim, the projection of each sample point in time in the 435-dimensional state space onto the vector defining the mean difference between the behavior of the group of animals that had received the highest dose of each drug and the saline treated control group in the same experiment, 〈*highest dose*–*control*〉 were calculated (cf. ref. 37). In this metric, the normal swimming behavior of the control group will on average correspond to the value zero whereas the average behavior displayed by the group of larvae treated with the highest dose will correspond to the value one, regardless of what type of behavioral changes that are induced by each specific drug (Fig. 5L–O *;* cross-validations of these data are summarized in Supplementary Table 3). It was evident that robust and stable dose-dependent alterations of swimming behavior was induced by each of the drugs evaluated, further corroborating the methodological approach. However, the changes observed in global swimming behavior involve complex combinations of kinematic features and therefore provide limited information on the specific physiological changes underlying the unique behavioral states. As a final step we therefore summarized to what extent the 435 different features were affected by the various pharmacological manipulations by plotting their relative contribution to the difference vector 〈*highest dose*–*control*〉 (Fig. 5P–S; in this representation the relative contribution of each feature is denoted by the distance from zero). Interestingly, and as noted for the example features above, both very basic changes in swimming behavior - such as the average speed and duration of bouts - and several more complex changes in the pattern of action sequencing - such as the relative frequency of observation of specific waiting times or bouts pairs – proved to be sensitive to the pharmacological manipulations. Hence, potentially allowing for also more in depth qualitative analyses of behavioral alterations.

## Discussion

The highly discretized swimming behavior of zebrafish larvae provides a unique opportunity to study patterns of action sequencing. It is however a significant experimental challenge to document and describe the motor repertoire of hundreds of freely behaving millimeter-size individuals. Through the development of an automated high-resolution tracking system we have here managed to overcome this complication and have characterized the swimming behavior with a greater precision in larger groups of zebrafish larvae than what has been achieved with previously existing systems. Based on these larger data sets it was possible to evaluate how well theoretical models of possible network mechanisms underlying behavioral transitions could reproduce actual swimming behavior. Most importantly, we could provide direct evidence for action sequencing which opens up for further studies of more complex aspects of motor control in this species. Finally, our results clearly show that zebrafish larvae are sensitive to pharmacological manipulations of motor control networks in the same way as mammals^38, 39^ and hint at an important role of the basal ganglia in the control of motor behavior given the clear sensitivity to monoaminergic manipulations with respect to sequencing of actions, as previously suggested based on related findings in lamprey^5, 9, 10, 40^.

An inherent limitation when using fish larvae instead of rodents in behavioral analyses is a somewhat reduced complexity in the motor repertoire. It is also important to keep in mind that environmental factors, including for example the size and color of the individual wells, can influence the behavior displayed by the larvae. At the same time, the choice of analytical procedures in the characterization of kinematic features is also an important factor, in itself. Previous studies have often focused on only a few kinematic parameters that have been averaged over predetermined time intervals, thus severely restricting the amount of information available^41^. The multivariate analyses of bout organization applied in the present study allowed for significantly more comprehensive descriptions of behavior, that could potentially be adapted for kinematic analyses also in other species^42^. Furthermore, it is interesting to note that if the complex sequencing of actions here discovered in zebrafish larvae reflects a shared functional role of the basal ganglia in vertebrates (cf. refs 32, 43, 44) this could make fish larvae a relevant model system for a whole range of disease conditions, including for example Parkinson’s disease and obsessive compulsive disorder. It could also pave the way for new ways to study genetic influences on behavior since zebrafish is a very widely used genetic model system.

From observations in patients and mammalian model systems of basal ganglia disease, it is known that a wide range of behavioral changes can be observed and that similar dysfunctions may occur as a consequence of exposure to certain drugs acting on the central nervous system (CNS). Such observations is the rationale for the use of behavioral analyses in the search for novel drug candidates. In this type of phenotypic screening procedures, a potential new drug can theoretically be identified on the basis of, either, (1) showing therapeutic-like effects restoring motor function in an animal model of a disease or, conversely, (2) mimicking the behavioral side-effects induced by a certain class of clinically used drugs. Laying a foundation for such screening methods, several experimental studies have been designed to pharmacologically target monoaminergic systems, bearing some similarities to the experiments in the current study^35, 45, 46^. Again however, because the level of detail in those kinematic descriptions are considerably lower, direct comparisons to the current data-set are less relevant. In this context, it is worth emphasizing that a higher level of detail is also a prerequisite to allow for meaningful comparisons to human motor symptoms. Various types of abnormal motor behaviors and motor dysfunctions can be categorized as for example: bradykinesia, dystonia, dyskinesia, chorea, stereotopies, ataxia, tremor, myoclonus etc. Clearly, this wide range of different types of motor symptoms cannot be accurately quantified by simple measures of the overall amount of motility displayed. Instead, it is the relative frequency by which certain behaviors are performed over others, or more detailed changes in in the organization of sub-components of the motor repertoire that need to be assessed in order to distinguish these types of abnormalities in the motor behavior. An example of a more specific type of motor dysfunction that may arise as a side-effect of pharmacological stimulation of monoaminergic systems or in association with obsessive compulsive disorder is movement stereotypies. So far, stereotypic behavior has mainly been studied mammals^47, 48^, but could now be effectively explored also in fish larvae using our developed technology. Thus methodological development of this kind, offering improved behavioral characterizations, could in the future have a significant impact outside the field of basic neuroscientific research – in particular, since zebrafish is rapidly becoming more frequently used for *in vivo* screening in drug discovery assays^49–52^.

Diseases affecting the CNS is a rapidly growing concern for societies all around the world. At the same time, the rate of progress in the search for new treatment options is currently disappointingly slow. It has been argued that a major reason behind this troublesome situation is the fact that early screening of new drug candidates has only to a limited extent been carried out in intact animals, while reduced model systems such as *in vitro* assays have so far shown low predictive value for clinical treatment of CNS disease^53^. On the other hand, assays based on *in vivo* screening have proven somewhat more successful in terms of the number of new drugs developed but the very high costs associated with large scale studies in standard laboratory animals like rats and mice unfortunately limits the practical value of this approach. Hence, simpler animal models have been evaluated with the aim of partially substituting experiments in mammals. In particular zebrafish larvae have attracted attention as a possible *in vivo* model, allowing for behavioral monitoring of relatively large groups of animals, in parallel, following pharmacological interventions.

Thus, taken together, if behavioral screening in zebrafish is to become a useful tool for drug development it is important to provide more sensitive techniques for screening of behavioral changes using a system for automatic tracking of swimming behavior. The sensitivity and reliability of our developed tracking system which was here evaluated under diverse experimental conditions, including pharmacological manipulations applying substances that are known to induce changes in the organization of behavior in humans, proves the feasibility of this approach. We therefore believe that the technology and biological findings presented herein should have important implications both for future studies of motor control systems and for researchers interested in improving the technology for *in vivo* screening of novel compounds in drug development.

## Methods

### Animals

This study was conducted in accordance with the national legislation of Sweden and the European Community guidelines for animal studies. All procedures were approved by the ethical committee in Malmö-Lund (Permit, M116-12).

The zebrafish larvae used in this study were from intercrosses of the wild-type AB strain. Embryos were collected and raised in a 14:10-hour light/dark cycle at 28.5 °C on petri dishes containing E3 embryo medium (5 mM NaCl, 0.17 mM KCl, 0.33 mM CaCl_2_, and 0.33 mM MgSO_4_) in an incubator to 5 days post-fertilization (dpf). At the age of 5 dpf, the larvae were transferred into 0.8 L aquaria and placed in a recirculating system held at 26 ± 1.5 °C (Aquaneering, Inc., San Diego, CA) where feeding was initiated. Larvae were fed with a commercial larval diet, ZM000 (ZM Fish Food & Equipment, Winchester, UK), three times daily until the age of 10 dpf. Behavioral experiments were conducted at 10 dpf (and 15 for MK-801 experiments). The age of the zebrafish larvae was chosen to ensure a developed blood-brain barrier, which is an important feature when evaluating potential pharmaceuticals for CNS diseases.

### Experimental setup and video recordings

Each individual experiment was performed using 48 zebrafish larvae, 2 × 24 well microtiter plates (Cat. No. 303002; Porvair Sciences, Leatherhead, UK) milled to a depth of 9 mm to reduce shadow artifacts and with white walls to increase contrast between larvae and background and to prevent larvae in adjacent wells from acting as visual stimuli. The behavioral chamber consisted of a 300 fps digital camera (Genie HM640, Teledyne DALSA, Waterloo, Canada) connected to a computer set up with video recording software (CamExpert v7.00.00.0912, Teledyne DALSA, Waterloo, Canada; Labview™ 2011 v11.0, National Instruments, Austin, TX. To maintain the environment in the wells at 28 °C the microtiter plates were placed parallel to each other in a water bath containing a temperature control unit (Neoheater 25 W thermostat, AQUAEL, Warsaw, Poland). The microtiter plates were positioned on top of a light box, containing LED strips (SMD5050 flexible infrared 850 nm tri-chip). Prior to each experiment, larvae were transferred to the microtiter plates containing 1 ml of E3 medium and all individuals were observed for abnormal swimming behavior and body deformities. Damaged individuals were removed and replaced. The experiments were performed in darkness (using IR-illumination). The zebrafish larvae were habituated for 45 min before the experiment. During the experiment, video recordings were obtained in 5 min time periods in order to be able handle the large amount of data (>5GB/min), creating brief interruptions (∼1 s) during data transfer to storage devices.

### Pharmacology

For the amphetamine experiments, larvae were analyzed in 4 treatment groups (0.1, 1.0, 10 μM and control). The experiment was performed in triplicate including in total 36 larvae per group. For drug preparation compound (D-amphetamine sulfate, 2813, Tocris Bioscience) was first dissolved in 100% dimethyl sulfoxide to generate a stock solution (10 mM). The stock solution was then diluted in E3 medium to 500 μM, 50 µM and 5 µM and 20 µL of these solutions were added to the wells. The control group was given 20 µL of a 5% DMSO solution corresponding to a final concentration of 0.1% DMSO in the well.

For the MK-801 experiments, larvae were analyzed in 4 treatment groups (1, 5, 20 μM and age matched control). The experiment was performed in duplicate including in total 24 larvae per group. For drug preparation compound ((+) MK-801 hydrogen maleate, M107) was first dissolved in 100% dimethyl sulfoxide to generate a stock solution (10 mM). The stock solution was then diluted in E3 medium to 1000 μM, 250 µM and 50 µM and 20 µL of these solutions were added to the wells. The control group was given 20 µL of a 5% DMSO solution corresponding to a final concentration of 0.1% DMSO in the well.

For the apomorphine experiments, larvae were analyzed in 8 treatment groups divided in a high dose (10, 25 and 50 μM) and a low dose (0.1, 0.2 and 0.5 μM) treatment paradigm. The high dose paradigm was performed in duplicate and the low dose paradigm in triplicate experiments including in total 24 and 36 larvae ﻿per group, respectively. For drug preparation compound (A4393, Sigma-Aldrich, St. Louis, MO) was first dissolved in 100% dimethyl sulfoxide to generate a stock solution (10 mM). The stock solution was then diluted in E3 medium to 2.5 mM, 1.25 mM, and 500 μM (high), or 25 µM, 10 µM, and 5 µM (low).

### Tracking of swimming behavior

The swimming behavior of the zebrafish larvae was analyzed by first tracking the positions of the larvae in video recordings by an automatic image analysis algorithm and then inputting the tracking results into the behavioral analysis algorithms described below. This section describes the automatic image analysis application.

Given a video sequence of zebrafish larvae recorded using a static camera and static lighting conditions, the static *background* is computed by modelling each pixel at each point in time as belonging to one of two Gaussian distributions, where the brighter distribution defines background and the darker distribution defines foreground. The distributions are estimated using a moving average estimation algorithm, described in the following. Background and foreground are modeled using normal distributions for each pixel with expected values *B* and variances *S* for the background and $\hat{B}$ and $\hat{S}$ for the foreground. The model parameters are initialized by drawing 100 randomly selected frames and computing the mean *M* and variance *V* of them for each pixel, then setting ${B}^{(0)}=M+\sqrt{V}$, ${\hat{B}}^{(0)}=M-\sqrt{V}$ and ${S}^{(0)}={\hat{S}}^{(0)}=V$. For 1000 additional randomly selected frames ${I}^{(k)}$, the probability that ${I}_{ij}^{(k)}\in N({B}^{(k)},{S}^{(k)})$ is compared to the probability that ${I}_{ij}^{(k)}\in N({\hat{B}}^{(k)},{\hat{S}}^{(k)})$. All pixels $(i,j)$ that are estimated to be more probable to belong to the background and the corresponding elements of *B* and *S* are updated with the updating coefficient *α* as follows

1 $${B}_{ij}^{(k+1)}=\alpha {B}_{ij}^{(k)}+(1-\alpha ){I}_{ij}^{(k)},$$

2 $${S}_{ij}^{(k+1)}=\alpha {S}_{ij}^{(k)}+(1-\alpha ){({B}_{ij}^{(k+1)}-{I}_{ij}^{(k)})}^{2}.$$

The foreground parameters ${\hat{B}}^{(k)}$ and ${\hat{S}}^{(k)}$ are updated analogously for the pixels that are estimated to be more probable to belong to the foreground. The resulting expected value of the background distributions define the background image.

Given an image $I$ and the estimated background image $B$, the difference image *D* = *B* − *I* is computed. Since the pixels corresponding to zebrafish larvae are darker than the background, *D* is an image in which higher values are more likely to correspond to zebrafish larvae. The larva head is assumed to be at the position of the maximal value (robustly measured given a Gaussian kernel of size 5 × 5 pixels) of *D* in each well. An initial value of the head position is given by finding the position of the maximum of *D* after applying a Gaussian filter. The initial value is improved to subpixel-precision by quadratic interpolation. Given the estimated head position, the tail of the larva is found by the positions of maximal intensity at 7 circles of increasing radii, centered at the head.

### Extraction of swim bouts

As previously shown, the zebrafish larvae appears to move in short bursts of movement (denoted *swim bouts*; see Fig. 1E,F). Therefore, an important task in the analysis of zebrafish larvae behavior is to segment the tracking data that corresponds to swim bouts.

This is done by estimating the speed of the larvae and defining the time intervals in which the larvae is moving faster than some threshold as swim bouts. In order to make the swim bout segmentation robust to oscillatory tracking errors, the following speed measure is used

3 $$v(t)=\mathop{min}\limits_{k}{v}_{k}(t),$$

where

4 $${v}_{k}(t)=\frac{{\parallel (x(t+k),y(t+k))-(x(t),y(t))\parallel }_{2}}{k}\,.$$

In other words, the average speeds over various time windows *k* are first estimated, and then the min value over all time windows is used in subsequent swim bout segmentation. In this particular application (framerate, resolution, fish size, error size, etc.), the time window sizes $k=1,12,24,48$ have been defined empirically.

An example of applying this robust speed function on tracking data is shown in Fig. 1F. An initial segmentation of swim bouts can be performed by simply applying a threshold value (${v}_{threshold}$) on the robust speed estimates, i.e. the points in time $t$ for which $v(t) > {v}_{threshold}$ are classified as swim bouts. As is common in this type of application and segmentation applications in general, there are sometimes uncertainties to resolve after applying the threshold and performing the initial segmentation. Firstly, initial swim bouts that are too short (<0.03 s) are removed and secondly, swim bouts that contain an estimated speed component that is too large (>5 m/s) are removed. Thus a set of swim bouts can be constructed, each on the form $({x}_{1k},{y}_{1k},\ldots ,\,{x}_{8k},{y}_{8k},k)$, where *k* is the frame number. In this notation, $({x}_{1k},{y}_{1k})$ corresponds to the head and $({x}_{8k},{y}_{8k})$ corresponds to the end of the tail.

### Classification of swim bouts

The process of classifying swim bouts as belonging to one of *K* classes is described here. First, swim bouts from a control experiment are normalized and subsampled. Secondly, the k-means algorithm is applied to separate the data into *K* groups and compute the group centers (i.e. the mean trajectories of the classes). Thirdly, a swim bout is classified as belonging to the class for which the distance from the corresponding mean trajectory to the normalized and subsampled swim bout is minimized.

To enable meaningful comparison of swim bouts, swim bouts are normalized with respect to starting position, direction and time. The tracking data of each swim bout is rotated and translated such that it starts at the origin at time zero and is facing towards the positive x-axis. In practice, this means that a rigid transformation *T* is sought such that the transformed head coordinate, ${\hat{x}}_{11}=T{x}_{11}$, of the first frame in the swim bout is at the origin and the transformed tail coordinates, ${\hat{x}}_{i1}=T{x}_{i1}$, $i=2,\ldots ,8$, lies approximately (minimizing the sum of squared distances) on the positive x-axis. Then all subsequent frames are transformed using the estimated *T*. In the current analyses, body curvature was not specifically analyzed. As a consequence, the tail positions were discarded after this step keeping solely the head position. Due to the variety in duration of swim bouts, a method for measuring similarities between tracking data of different lengths is necessary. This is provided by subsampling each swim bout by *M* = 20 (empirically defined) points that are equidistant in space, which effectively removes the time-dependency of the space dimensions while keeping it in the time-dimension. Note that the velocity information is not removed by this interpolation since the time-dimension is kept intact. Thereby, each swim bout is transformed to the normalized and subsampled form $\{({\hat{x}}_{k},{\hat{y}}_{k},{t}_{k}),k=1,\ldots ,M\}$ from which they are compared.

The trajectories of *N* normalized and subsampled swim bouts are stacked in a matrix

5 $$X=[{X}_{1}\,\cdots \,{X}_{N}\,],$$

where each *X*_*i*_ is the column stacked coordinates from swim bout $i$, i.e.

6 $${X}_{i}={[{x}_{1}{y}_{1}{t}_{1}\cdots {x}_{M}{y}_{M}{t}_{M}]}^{{\rm{T}}}.$$

To enable meaningful comparisons in the multidimensional space of different quantities (time and space), the matrix *X* is normalized as follows. All x-coordinates are normalized by subtracting the mean and dividing by the estimated standard deviation of all measured x-coordinates. The y and t-dimensions are normalized analogously.

The k-means algorithm with the Euclidean norm as metric is then applied on the normalized *X* to separate the data into *K* groups, and the mean trajectory of each group is then computed and reshaped back to the form $\{({x}_{k},{y}_{k},{t}_{k}),k=1,\ldots ,M\}$. The created mean trajectories are subsequently used for classification of swim bouts by measuring the Euclidean distance from each normalized and subsampled swim bout to the mean trajectories. A swim bout is then classified as belonging to the class with the closest mean trajectory. An example of estimated mean trajectories for *K* = 15 is shown in Fig. 2E,F, and the distribution of the population of classes is shown in Fig. 2G.

### Extraction of kinematic features and higher order organization features

The definitions of the kinematic features presented in Fig. 2B–D are introduced here. The distance of a swim bout is defined as the sum of Euclidean distances between all consecutive pairs of frames in the swim bout, i.e.

7 $$d=\sum _{k=1}^{n-1}{\parallel ({x}_{k+1},{y}_{k+1})-({x}_{k},{y}_{k})\parallel }_{2}.$$

Note that to increase robustness to noise, $({x}_{k},{y}_{k})$ is pre-filtered using a median filter of length 5 (empirically defined).

The duration of a swim bout is defined as the length of the interval in which the larva travels from 5% of the total distance to 95% of the total distance, where the method of measuring distance introduced above is used. The angular change of a swim bout is defined as the second encountered extreme angle minus the first encountered extreme angle, i.e. a swim bout in which the minimal angle −34 degrees is attained at *t* = 0.15 *s* and the maximal angle +13 degrees at *t* = 0.05 *s* is considered to have an angular change of −47 degrees.

The bout classes introduced in the previous subsection are also used for analyzing swimming behavior. Tracking data for each zebrafish larva is converted to the form $\{({c}_{k},{t}_{0,k},{t}_{1,k}),k=1,\ldots ,N\},$ where ${c}_{k}$ is the estimated class of swim bout *k*, ${t}_{0,k}$ and ${t}_{1,k}$ are the start and end times of the swim bout, respectively, and $N$ is the number of swim bouts. The vector ${w}_{k}={t}_{0,k+1}-{t}_{1,k}$ defines the inter-bout waiting times for $k=1,\ldots ,N$, and the distribution of waiting times for a control experiment is presented in Fig. 3A. A threshold ${w}_{short}$ is applied on the vector $w$ to classify each waiting time as short or long. Here, the threshold is individually set to the median of ${w}_{k}$ for each larva and thus creates equally populated groups of short and long waiting times for each larva. Higher order organization of waiting times can be analyzed by considering the conditional probability that ${w}_{k}$ is short given that the previous *N* waiting times have been short, i.e.

8 $${p}_{short}^{N}=P({w}_{k} < {w}_{short}|{w}_{k-1} < {w}_{short},\ldots ,{w}_{k-N} < {w}_{short}).$$

Figure 3B shows an estimate of ${p}_{short}^{N}$ as a function of *N* together with the theoretical probability for the memoryless process ${w}_{k}$.

The sequencing of swim bouts is analyzed in Fig. 4B,C. The probability that an observed swim bout subsequence $({c}_{k},{c}_{k-1})$ consists of movement class $A$ followed by movement class $B$, i.e. $P({c}_{k}=B,{c}_{k-1}=A)$, is shown for a few examples of (*A*, *B*) in Fig. 4B. Also the probability that both swim bouts in a subsequence $({c}_{k},{c}_{k-1})$ are of the same class is shown as (*X*, *X*) in the figure. The conditional probability that a swim bout is of the same class as all the *N* previous swim bouts, given that all *N* previous are of the same class, is presented in Fig. 4C.

### Dose-response tests

The behavioral alterations induced by drug administration is investigated by quantizing the kinematic features and higher order organization features presented above. Each of the features that follows is evaluated independently for each zebrafish larva and each time period. The values given by the following features are entered into a *feature matrix* F of size M × N × K, where M is the number of unique features, N is the number of time bins and K is the number of fish. First, the distributions of swim bout duration, distance, turning, inter-swim bouts waiting times and swim bouts classes are estimated and normalized in 10, 10, 20, 15 and 10 bins, respectively. The sequencing of inter-swim bout waiting times is represented by all possible conditional probabilities of order 5 or lower. The sequencing of swim bout classes is entered as the conditional probabilities of order 2.

For each drug type, the mean vector from the control group to the highest group was computed and subsequently used to define the dose-response vector. For each dose in the drug group and each time bin, the mean vector was computed and projected on the dose-response vector. The results are shown in Fig. 5L–O.

### Statistical tests

Statistical tests used are specified in the main text in the context they were used.

### Data availability

The datasets generated during and/or analyzed during the current study are available from the corresponding author on reasonable request.

## Electronic supplementary material

Supplementary Information

Supplementary Video
