# Supplementary material for: Action sequencing in the spontaneous swimming behavior of zebrafish larvae - implications for drug development
Source: Sci Rep. 2017 Jun 9;7:3191. doi: 10.1038/s41598-017-03144-7 (PMC5466685; doi:10.1038/s41598-017-03144-7)
Supplement: Supplementary file 1 — Supplementary Information [file 41598_2017_3144_MOESM1_ESM.pdf]

Supplementary Information

Action sequencing in the spontaneous swimming behavior of zebrafish larvae - implications for drug development

Tobias Palmér [+,1,2], Fredrik Ek [+,3], Olof Enqvist[,4], Roger Olsson[§,3], Kalle Åström[§,2] and Per Petersson[§,1, *]

+) shared first authorship, §) shared senior authorship, *) corresponding author

Affiliations: 1) Integrative Neurophysiology and Neurotechnology, NRC, Department of Experimental Medical Sciences, Lund University, Sweden. 2) Mathematics, Centre for Mathematical Sciences, Faculty of Engineering, Lund University, Sweden. 3) Chemical Biology & Therapeutics, Department of Experimental Medical Sciences, Lund University, Sweden. 4) Department of Signals and Systems, Chalmers University of Technology, Sweden

Correspondence: Per.Petersson@med.lu.se

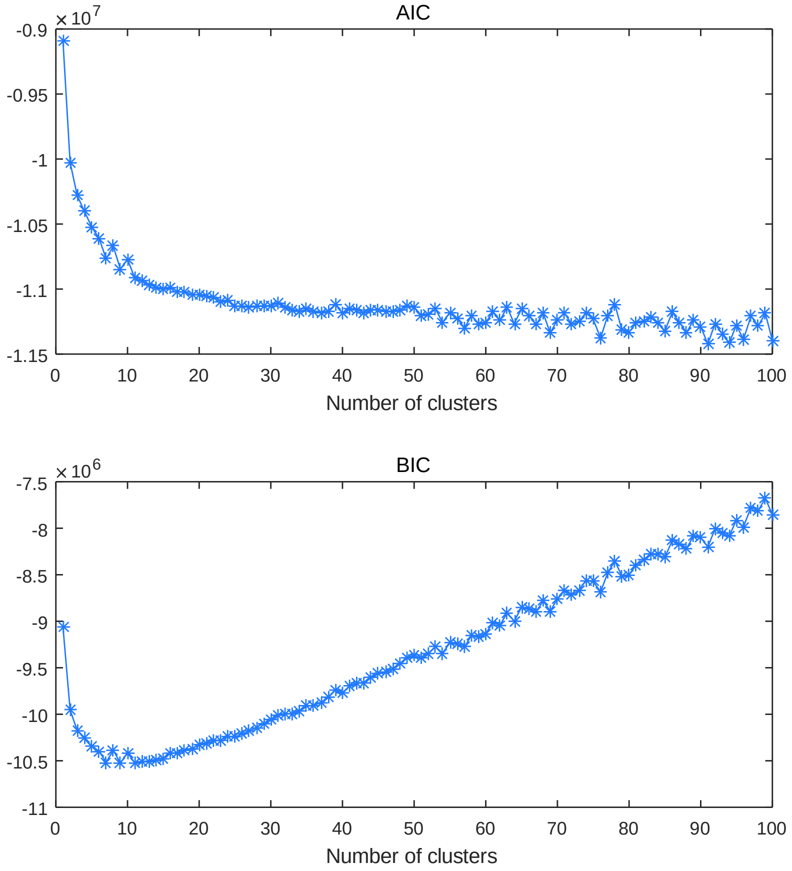

Supplementary Figure 1

Plots of the Akaike Information Criterion (AIC) and Bayesian Information Criterion (BIC) values used for deciding the number of clusters to use for the clustering of swim bouts.

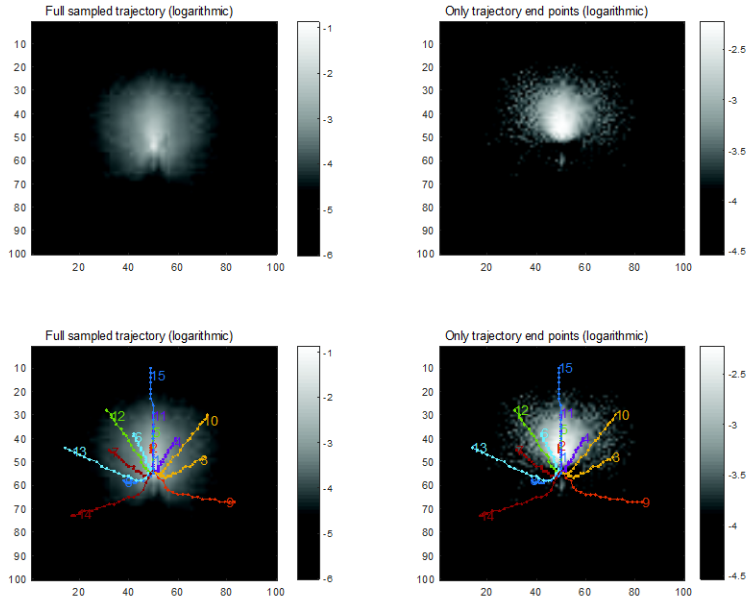

Supplementary Figure 2

Logarithmic density images of the data used for defining the swim bout classes. The x and y-coordinates of all points on the subsampled trajectories of normalized swim bouts (as described in the text) are used in the left column, and only the end points are used in the right column. The top row shows only the logarithmic density images while the bottom row show the same images with the computed swim bout-classes superimposed.

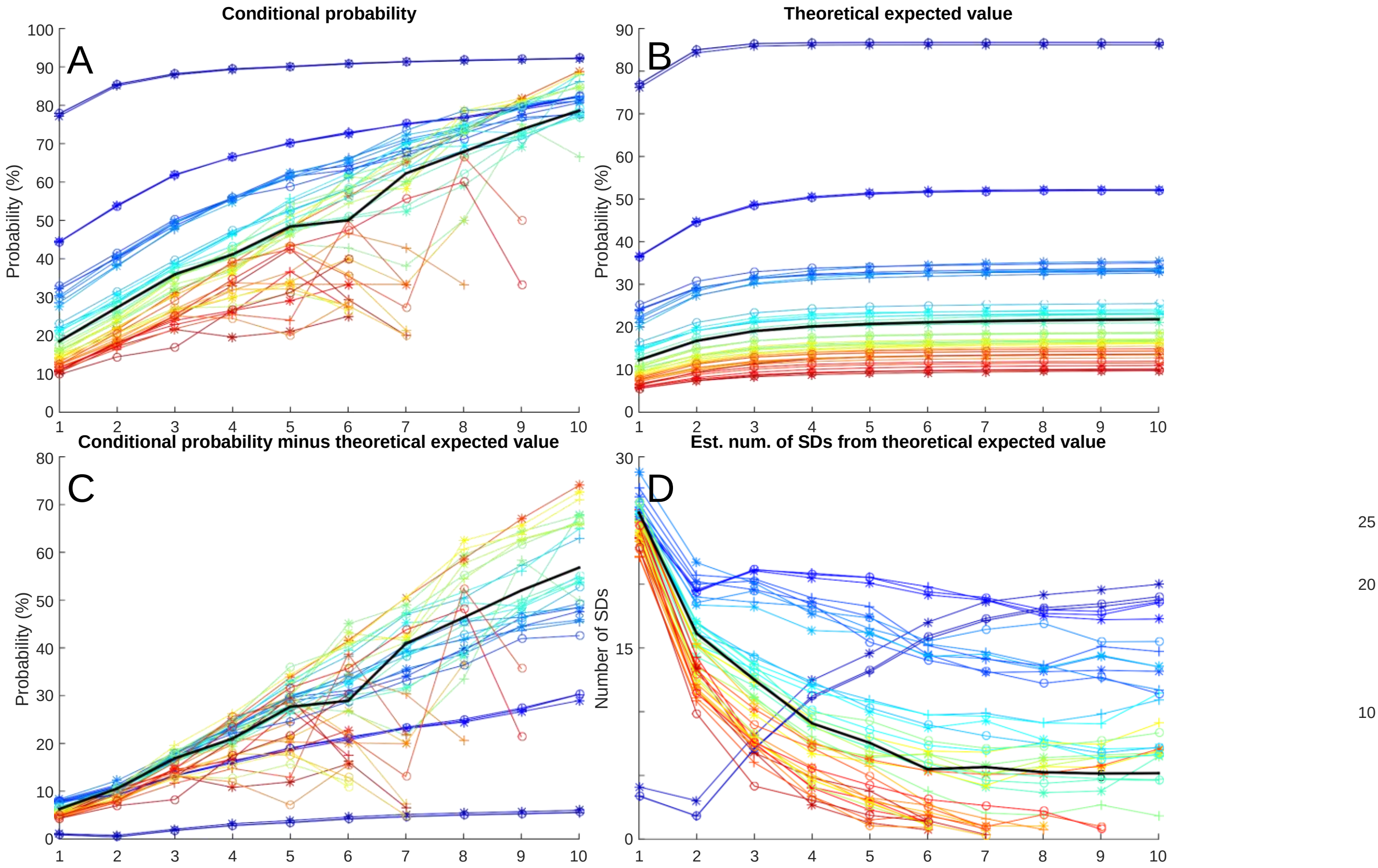

Supplementary Figure 3

Control analyses showing that the result that bouts of the same type are often repeated (see Figure 4) is not sensitive to the exact choice of number of trajectory sample points or bout classes (colors represent few (blue; n=2) to many (red; n=20) clusters and symbols for the three lines of each color (*, +, o) represent 10/20/30 sample points, respectively. Black line denotes the data presented in Figure 4C (20 samples and 15 bout clusters). A) Conditional probabilities of bout repetitions estimated from observed data, B) Theoretical probabilities assuming no memory of previous bout type, C) Difference between observed and theoretical probabilities (i.e. A-B), and D) The differences observed in C, in terms of number of standard deviations (Z-score; estimated from variance in A). Note that too few clusters makes it highly likely to repeat the same bout type by chance and that too many clusters makes in unlikely to observe a sufficient number of events to compare observed data to chance level. Importantly, for all selections of sample and cluster numbers identical bout types are repeated above chance level.

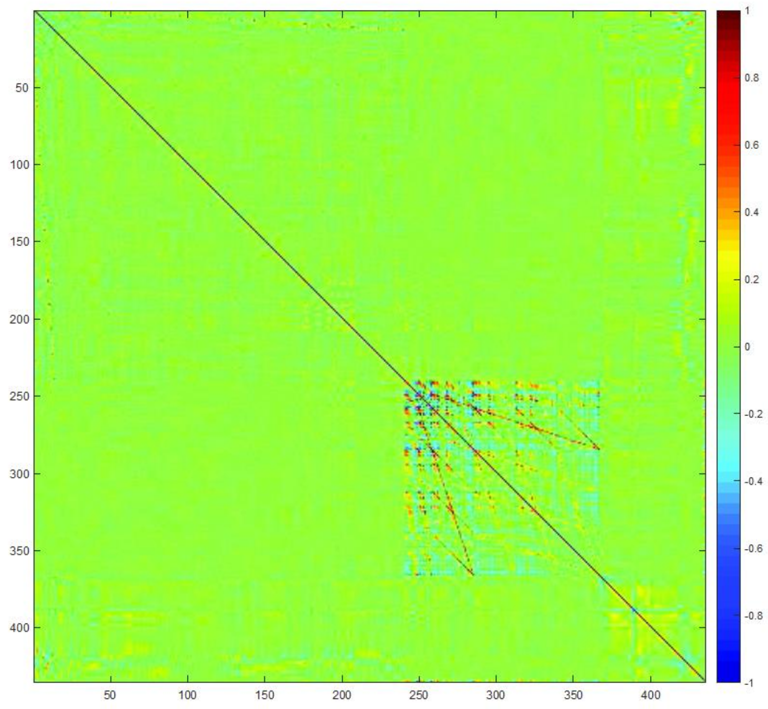

Supplementary Figure 4

The estimated correlation matrix of the 435-dimensional feature matrix indicates that the interdependence of the selected features is relatively limited.

Supplemental Video - Video sequence providing a 3D representation of the bout trajectories shown in Figure 2E-F.

Supplementary Table 1 A-E

Quantitative comparison of goodness-of-fit to experimental data for five different models (cf. Figure 2 and 3) in terms of Negative log-likelihood, Akaike information criterion (AIC) and Bayesian information criterion (BIC)

A) Duration

Estimated O-U drive: 0.0069

Name	Negative log-likelihood	Number of parameters	AIC	BIC
O-U	-57857.346662	2	-115710.693323	-115693.774444
Random walk	-24993.089016	1	-49984.178031	-49975.718592
Poisson	-55242.437710	2	-110480.875419	-110463.956540
Exponential	-34998.498254	1	-69994.996508	-69986.537068
Normal	-52862.555626	2	-105721.111253	-105704.192374

Estimated O-U drive: 0.0021

Name	Negative log-likelihood	Number of parameters	AIC	BIC
O-U	42000.893583	2	84005.787166	84022.706045
Random walk	58244.798334	1	116491.596668	116500.056108
Poisson	48438.886804	2	96881.773607	96898.692486
Exponential	50680.659187	1	101363.318374	101371.777813
Normal	52505.896090	2	105015.792181	105032.711060

C) Positive angles

Estimated O-U drive: 0.0007

Name	Negative log-likelihood	Number of parameters	AIC	BIC
O-U	3552.898354	2	7109.796708	7125.564298
Random walk	6558.035232	1	13118.070464	13125.954259
Poisson	11229.373359	2	22462.746717	22478.514307
Exponential	5353.108535	1	10708.217070	10716.100865
Normal	14683.160193	2	29370.320386	29386.087975

D) Negative angles

Estimated O-U drive: 0.0006

Name	Negative log-likelihood	Number of parameters	AIC	BIC
O-U	4846.520811	2	9697.041623	9712.307865
Random walk	7094.813967	1	14191.627935	14199.261056
Poisson	10062.976522	2	20129.953043	20145.219286
Exponential	5928.726458	1	11859.452916	11867.086037
Normal	12507.930225	2	25019.860450	25035.126692

E) Inter-bout waiting time

Estimated O-U drive: 0.0001

Name	Negative log-likelihood	Number of parameters	AIC	BIC
O-U	80715.916666	2	161435.833331	161452.717266
Random walk	81342.720121	1	162687.440241	162695.882209
Poisson	104435.273909	2	208874.547818	208891.431753
Exponential	83921.485699	1	167844.971397	167853.413364
Normal	117529.903733	2	235063.807466	235080.691401

Supplementary Table 2

Feature name	Feature description for a given zebrafish larvae and a time interval.	Number of values
Distribution of bout classes	The fraction of swim bouts that are of class X.	1-15
Distribution of second order bout classes chains	The fraction of pairs of consecutive swim bouts where the first is of class X and the second of class Y.	16-240
Distribution of inter-bout waiting times	The fraction of waiting times that are among the k/N and (k+1)/N shortest waiting times (by the all-time waiting time distribution of the current zebrafish larvae). N is the number of bins and k goes from 0 to N-1. Here, N=9 is used.	241-249
Distribution of second, third and fourth order inter-bout waiting time chains	The fraction of (two/three/four)-pairs of consecutive waiting times where the first is of type X, the second of type Y, etc. Here the waiting times are binned by N = 3.	250-366
Distribution of swim bout durations	The fraction of swim bouts with durations between $0.05k$ seconds and $0.05(k+1)$ seconds, where k goes from 0 to 19. Longer swim bouts are placed in bin number 21.	367-387
Distribution of swim bout distances	The fraction of swim bouts with distances between $0.1k$ mm and $0.1\ (k+1)$ mm, where k goes from 0 to 19. Longer swim bouts are placed in bin number 21.	388-408
Distribution of swim bout cumulative turning	The fraction of swim bouts with cumulative turning between $15k$ degrees and $15(k+1)$ degrees, where k goes from -12 to 11. Swim bouts with cumulative turning less than -180 degrees are placed in bin number 1 and swim bouts with cumulative turning greater than 180 degrees are placed in bin number 26.	409-434
Number of bouts per second	The number of bouts per second.	435

Supplementary Table 3 A-D

Cross-validation of dose response data shown in Figure 5M-P. Half of the data set was used to construct the <*highest dose – control*> vector and the remaining part of the data set was projected onto this vector.

The measures of separation used in the tables are defined as follows.

1. Ratio of highest dose projections significantly larger ($p<0.01$) than control.

2. Ratio of highest dose projections significantly larger ($p<0.01$) than the lowest dose (excluding control).

3. Ratio of highest dose projections significantly larger ($p<0.01$) than the middle dose.

4. Ratio of highest dose projections significantly larger ($p<0.01$) than all other doses (including control) simultaneously.

A) Dose response vectors defined on subset 1 and evaluated on subset 1

	AMP 10 µM	APO 0.5 µM	APO 50µM	MK 20 µM
1	100.0%	100.0%	83.3%	100.0%
2	100.0%	100.0%	16.7%	100.0%
3	100.0%	83.3%	33.3%	16.7%
4	100.0%	83.3%	16.7%	16.7%

B) Dose response vectors defined on subset 1 and evaluated on subset 2

	AMP 10 µM	APO 0.5 µM	APO 50 µM	MK 20 µM
1	100.0%	100.0%	33.3%	100.0%
2	100.0%	50.0%	0.0%	100.0%
3	100.0%	16.7%	0.0%	33.3%
4	100.0%	16.7%	0.0%	33.3%

C) Dose response vectors defined on subset 2 and evaluated on subset 1

	AMP 10 µM	APO 0.5 µM	APO 50 µM	MK 20 µM
1	100.0%	100.0%	16.7%	100.0%
2	100.0%	100.0%	33.3%	66.7%
3	100.0%	0.0%	0.0%	16.7%
4	100.0%	0.0%	0.0%	16.7%

D) Dose response vectors defined on subset 2 and evaluated on subset 2

	AMP 10 µM	APO 0.5 µM	APO 50 µM	MK 20 µM
1	100.0%	100.0%	50.0%	100.0%
2	100.0%	100.0%	66.7%	100.0%
3	100.0%	83.3%	16.7%	50.0%
4	100.0%	83.3%	0.0%	50.0%

[Drug abbreviations: AMP=amphetamine; APO=apomorphine; MK=MK-801]
